# Placenta and fetal brain share a neurodevelopmental disorder DNA methylation profile in a mouse model of prenatal PCB exposure

**DOI:** 10.1016/j.celrep.2022.110442

**Published:** 2022-03-01

**Authors:** Benjamin I. Laufer, Kari Neier, Anthony E. Valenzuela, Dag H. Yasui, Rebecca J. Schmidt, Pamela J. Lein, Janine M. LaSalle

**Affiliations:** 1 Department of Medical Microbiology and Immunology, School of Medicine, University of California, Davis, Davis, CA 95616, USA; 2 UC Davis Genome Center, University of California, Davis, Davis, CA 95616, USA; 3 MIND Institute, School of Medicine, University of California, Davis, Sacramento, CA 95817, USA; 4 Perinatal Origins of Disparities Center, University of California, Davis, Davis, CA 95616, USA; 5 Department of Molecular Biosciences, School of Veterinary Medicine, University of California, Davis, Davis, CA 95616, USA; 6 Department of Public Health Sciences, School of Medicine, University of California, Davis, Davis, CA 95616, USA; 7 Present address: Department of OMNI Bioinformatics, Genentech, Inc., South San Francisco, CA 94080, USA; 8 These authors contributed equally; 9 Lead contact

## Abstract

Polychlorinated biphenyls (PCBs) are developmental neurotoxicants implicated as environmental risk factors for neurodevelopmental disorders (NDDs). Here, we report the effects of prenatal exposure to a human-relevant mixture of PCBs on the DNA methylation profiles of mouse placenta and fetal brain. Thousands of differentially methylated regions (DMRs) distinguish placenta and fetal brain from PCB-exposed mice from sex-matched vehicle controls. In both placenta and fetal brain, PCB-associated DMRs are enriched for functions related to neurodevelopment and cellular signaling and enriched within regions of bivalent chromatin. The placenta and brain PCB DMRs overlap significantly and map to a shared subset of genes enriched for Wnt signaling, Slit/Robo signaling, and genes differentially expressed in NDD models. The consensus PCB DMRs also significantly overlap with DMRs from human NDD brain and placenta. These results demonstrate that PCB-exposed placenta contains a subset of DMRs that overlap fetal brain DMRs relevant to an NDD.

## In brief

Exposure to polychlorinated biphenyls is a risk factor for a neurodevelopmental disorder. In a mouse model of exposure to a human-relevant mixture, Laufer et al. utilize WGBS to profile DNA methylation within placenta and fetal brain. Both tissues display shared alterations at regions related to neurodevelopment and autism spectrum disorders.

## Graphical Abstract

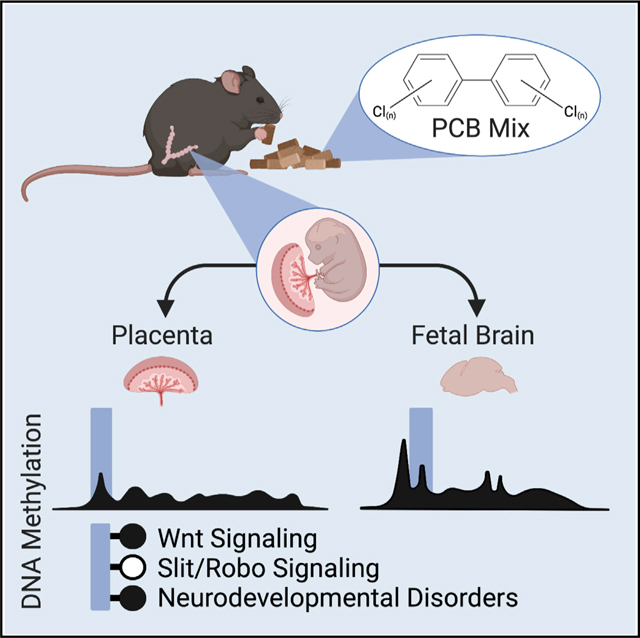

## INTRODUCTION

Polychlorinated biphenyls (PCBs) are a class of 209 structurally related congeners that were manufactured in the United States beginning in 1929 (Grimm et al., 2015). PCBs were manufactured as a mixture of congeners (e.g., Aroclor) and widely used in electrical equipment, primarily as coolants and insulating fluids for transformers and capacitators and as stabilizers in a number of commercial products, including paints and caulking. PCB production was banned in 1979 due to concerns about their environmental persistence and human cancer risk (Grimm et al., 2015). Despite the ban, legacy PCBs persist in the environment, and contemporary PCBs not present in the Aroclor mixtures are produced as a byproduct of current pigment and dye production used in paints and plastics (Grossman, 2013; Hu and Hornbuckle, 2010; Kostyniak et al., 2005; Martinez et al., 2012; Sjödin et al., 2014). Contemporary PCBs are detected in both indoor and outdoor air and in human food products (Chen et al., 2017; Hu et al., 2008; Thomas et al., 2012). Due to their persistent and lipophilic nature, legacy PCBs have accumulated in the marine food chains of the Great Lakes and the Artic, where they place the Indigenous Peoples at elevated risk for exposure (Brown et al., 2018; Hoover et al., 2012; Rawn et al., 2017). Finally, there is documented widespread exposure of humans to PCBs in a number of cities (Hens and Hens, 2017).

Prenatal exposure to PCBs can cause developmental neurotoxicity and is considered an environmental risk factor for various neurodevelopmental disorders (NDDs), including autism spectrum disorders (ASDs) (Klocke and Lein, 2020; Klocke et al., 2020; Panesar et al., 2020). Epigenetic mechanisms are involved in NDDs and have been associated with prenatal PCB exposure (Keil and Lein, 2016). Epigenetics refers to molecular mechanisms that regulate gene expression profiles related to development and tissue specificity. They do not require an alteration to DNA sequence, although they are heritable in dividing cells and have specialized regulatory roles in post-mitotic neurons. Examples of epigenetic mechanisms include DNA methylation, histone post-translational modifications, and non-coding RNA species. Specifically, altered DNA CpG methylation has been associated with PCB exposure and NDDs (Keil and Lein, 2016). Furthermore, PCB 95 levels are associated with a gene by environment interaction in a syndromic form of ASD caused by a chromosomal duplication (Dup15q), which is characterized by a global reduction in DNA methylation levels and enrichment for differential methylation at neurodevelopmental genes (Dunaway et al., 2016; Mitchell et al., 2012).

Differential placental DNA methylation has been separately associated with both PCB exposure and NDDs. As the maternal-fetal interface, the placenta is the organ responsible for removing toxicants; however, PCBs are capable of crossing the placental barrier and can also directly impact the placenta (Correia Carreira et al., 2011; Gingrich et al., 2020). In humans, term placenta is accessible at birth and characterized by a distinct DNA methylome with global hypomethylation and large partially methylated domains (PMDs), similar to the cancer methylome (Schroeder et al., 2011, 2013). Human term placenta is therefore a potentially rich source of epigenetic biomarkers for prenatal exposures, such as PCBs. PCB exposure was previously shown to be associated with differential methylation at select CpG sites in human placenta in an array-based approach (Ouidir et al., 2020). A low-pass, whole-genome bisulfite sequencing (WGBS) approach analyzing human placenta samples from the prospective high-risk MARBLES ASD cohort demonstrated that DNA methylation profiles distinguished ASD from control placenta and the top differentially methylated region (DMR) mapped to *CYP2E1* (Zhu et al., 2019). Notably, *CYP2E1* plays a key role in the metabolism of PCBs (Chen et al., 2018; Hu et al., 2020; Liu et al., 2017; Uwimana et al., 2019). Furthermore, in the MARBLES cohort, PCBs were detected in the serum of the pregnant mothers at levels that were experimentally shown to impact neurodevelopmental processes in model systems (Sethi et al., 2017a, 2019). When C57BL/6J mice were prenatally exposed to the MARBLES PCB mixture at 0.1 or 1.0 mg/kg/day through maternal diet, postnatal brain (~1 month old) showed PCB levels comparable to those reported in human samples (Lanting et al., 1998; Mitchell et al., 2012), coincident with changes in the dendritic morphology of hippocampal and cortical pyramidal neurons and changes in ASD-relevant behaviors (Keil Stietz et al., 2021; Sethi et al., 2021).

The objective of the research presented in this manuscript was to examine the effect of prenatal PCB exposure on placental and fetal brain DNA methylation profiles from the same mice in a human-relevant exposure model and to determine whether any regions in the placental methylome can serve as predictors of brain DNA methylation.

## RESULTS

### Global CpG methylation profiles are consistent with DMR profiles of PCB exposure

**Figure 1.**
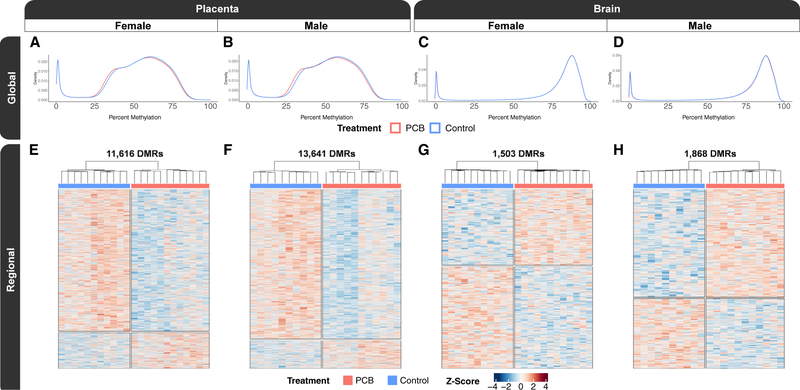
Sex-stratified global and regional DNA methylation profiles of placenta and fetal brain from mice with prenatal PCB exposure (A–D) Density plots of smoothed single CpG methylation values from (A) female placenta, (B) male placenta, (C) female brain, and (D) male brain. (E–H) Heatmaps of hierarchal clustering of the *Z* scores of regional smoothed methylation values for DMRs identified from pairwise comparisons of (E) female placenta, (F) male placenta, (G) female brain, and (H) male brain. A total of 44 placenta and 44 fetal brain methylomes were generated from PCB-exposed GD18 males (n = 11) and females (n = 12) and matched vehicle control males (n = 10) and females (n = 11).

To test the hypothesis that prenatal PCB exposure alters DNA methylation profiles in matched placenta and fetal brain, we generated a total of 44 placenta and 44 fetal brain methylomes (Data S1) from PCB-exposed (1.0 mg/kg/day) GD18 males (n = 11) and females (n = 12) and matched vehicle control males (n = 10) and females (n = 11). The methylomes were profiled by a low-pass WGBS approach that assayed ~20 million CpGs, which is ~90% of all CpG sites in the mouse genome. The global methylomes recapitulated known tissue-specific profiles (Schroeder et al., 2015). Specifically, both the female and male placental methylomes (Figures 1A and 1B) were hypomethylated when compared with their respective brain methylomes (Figures 1C and 1D). In placenta, there was significant global CpG hypomethylation (−1%; p ≤ 0.01) in both PCB-exposed females and males when compared with sex-matched controls. Placentas from PCB-exposed females had a global CpG methylation level of 51.9% and control females had a level of 52.8%, while PCB-exposed males had a level of 50.5% and control males had a level of 51.5%. In brain, the effects on global methylation were sex specific, as PCB-exposed males uniquely showed significant global hypermethylation when compared with vehicle male controls (0.1%; p = 0.006). Brains from PCB-exposed and control females both had a global CpG methylation level of 76.0%, while brains from PCB-exposed males had a level of 76.2% and control males had a level of 76.1%. In summary, placenta displayed PCB-associated global CpG hypomethylation in both sexes, while only males displayed global CpG hypermethylation in the brain, which had an effect size that was one order of magnitude less than the placental differences (Figure S1).

These differences in global CpG methylation levels were also consistent with finer resolution DMR analyses, which detected significant (empirical p < 0.05) locus-specific differences in DNA methylation that distinguished PCB-exposed from matched control (Table 1; Data S2). In addition, each pairwise DMR comparison provided background regions with a similar genomic context to the DMRs (gene length and CpG content) that were utilized in most of the downstream enrichment testing to control for genomic context. In placenta, PCB-exposed females displayed a profile of 11,616 DMRs (Figure 1E), which were identified from 210,247 background regions, and on average, the DMRs were 1,048 bp long and contained 12 CpGs. The placenta of PCB-exposed males displayed a profile of 13,641 DMRs (Figure 1F), which were identified from 243,350 background regions, and on average, the DMRs were 1,139 bp long and contained 12 CpGs. In brain, PCB-exposed females displayed a profile of 1,503 PCB DMRs (Figure 1G), which were identified from 28,562 background regions, and on average, the DMRs were 568 bp long and contained 11 CpGs. Brain from PCB-exposed males displayed a profile of 1,868 DMRs (Figure 1H), which were identified from 37,417 background regions, and on average, the DMRs were 608 bp long and contained 10 CpGs. In addition to placenta containing approximately an order of magnitude more PCB DMRs than brain, there was a hypomethylation skew in the placental DMRs from both sexes, where 81% of female and 85% of male placental DMRs were hypomethylated. There was a skew toward hypermethylation in only the male brain, where 43% of female and 61% of male brain DMRs were hypermethylated. The brain DMRs were approximately half the length of the placenta DMRs, on average, despite containing almost the same number of CpGs. In contrast to treatment group, litter did not overinfluence hierarchical clustering of individual methylation levels within the DMRs, while tissue and sex had a stronger effect on the global methylome than either litter or PCB exposure (Figure S2).

Next, to investigate the impact of prenatal PCB exposure on gene expression, we also generated matched RNA sequencing (RNA-seq) data for all samples. Similar to the methylomes, the placental transcriptomes displayed a larger PCB effect than the brain transcriptomes (Figures S3A–S3D). The nominally significant (p < 0.05) sex-stratified differentially expressed genes (DEGs) distinguished PCB-exposed placenta and brain (Figures S3E–S3H). However, the DEG profile was not as robust as the DMR profile (Data S3).

### Prenatal PCB exposure DMRs are functionally enriched for cell signaling and neurodevelopmental processes

**Figure 2.**
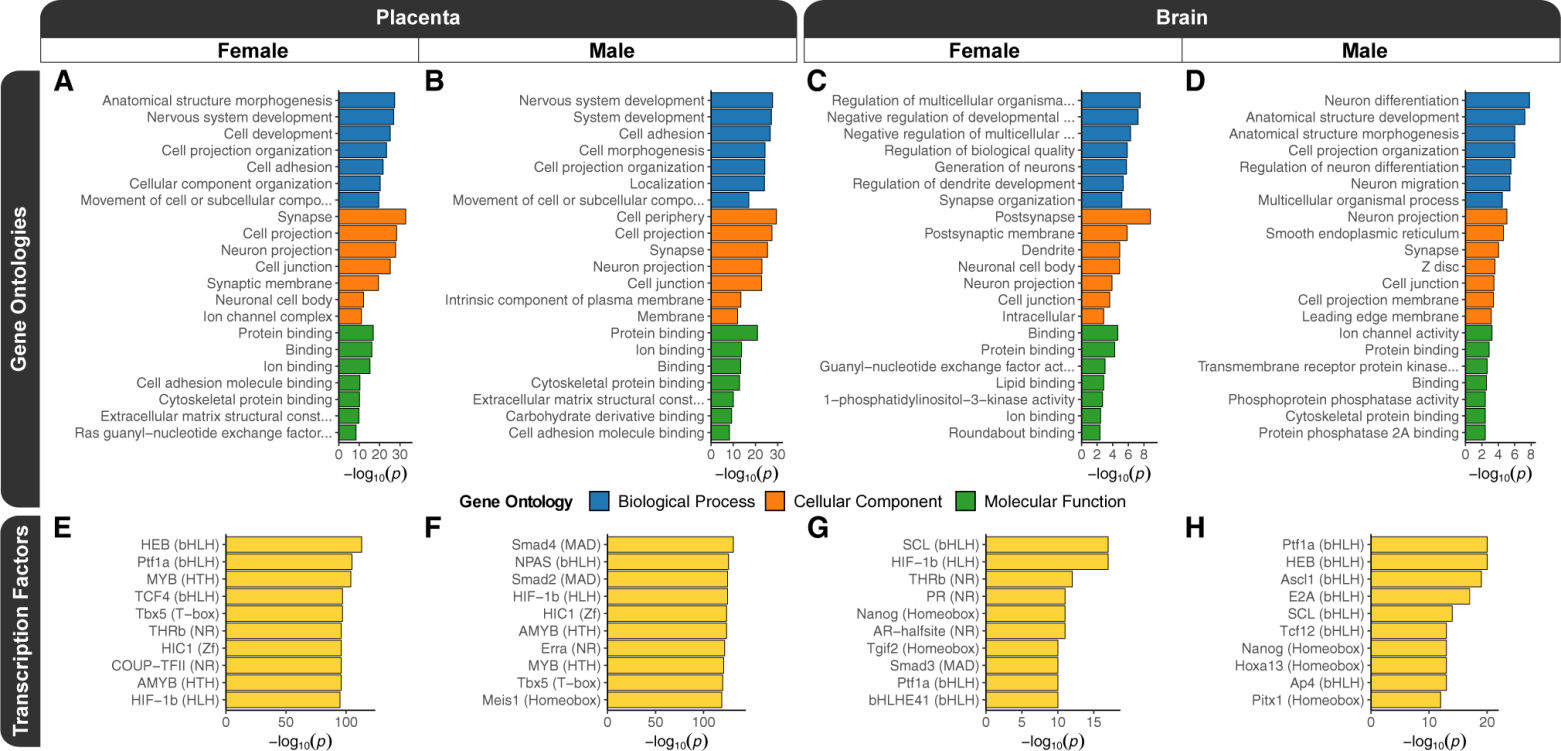
Functional enrichment testing results for sex-stratified prenatal PCB exposure DMRs from placenta and fetal brain (A–D) Top slimmed significant (p < 0.05) GO enrichment results for DMRs from pairwise comparisons of (A) female placenta, (B) male placenta, (C) female brain, and (D) male brain. (E–H) The most significant (q < 0.01) transcription factor motif enrichments for pairwise comparisons of (E) female placenta, (F) male placenta, (G) female brain, and (H) male brain. The motif family is indicated in parentheses.

To test the hypothesis that the prenatal PCB exposure DMRs occur in functional regions of developmentally relevant genes, we performed a series of enrichment testing analyses. We examined the biological relevance of the genes mapping to DMRs, relative to genes mapping to their background regions, through gene ontology (GO) analyses. The top significant (p < 0.05) slimmed GO enrichments were biological process terms related to neurodevelopment and development, cellular component terms related to the synapse and cell membrane, and molecular function terms related to ion and protein binding as well as cellular signaling (Figures 2A–2D; Data S2). Furthermore, a number of terms passed a more stringent significance (family-wise error rate [FWER] < 0.05) threshold, which was based on 1,000 random sets from samplings of the background regions, and the placenta showed a stronger enrichment. In female placenta, these terms were protein binding, anatomical structure morphogenesis, nervous system development, binding, cell development, ion binding, synapse, cell projection organization, and cell projection. In male placenta, the terms were protein binding, nervous system development, system development, cell adhesion, cell periphery, cell morphogenesis, cell projection organization, localization, and ion binding. In female brain, the only term was postsynapse, and there were none that passed this stringency threshold in male brain. The GO terms shared between sexes and tissues were consistent with the top significant (q < 0.1) protein analysis through evolutionary relationships (PANTHER) pathway enrichments, which were related to glutamate signaling, axon guidance mediated by Slit/Robo, integrin signaling, and endothelin signaling (Figures S4A–S4D; Data S2). Finally, the significant (p < 0.05) GO terms for the DEGs were enriched for ubiquitination and the proteasome, developmental signaling pathways, epigenetic mechanisms and gene regulation, apoptosis and cellular stress, the cytoskeleton and cell adhesion, and immunity (Figures S3I–S3L; Data S3).

### Prenatal PCB exposure DMRs are enriched for the motifs of developmental transcription factors

To examine the functional gene regulatory relevance of the PCB DMRs relative to their background regions, they were tested for known transcription factor binding motif enrichments using two different approaches. Notably, the GO term enrichments for developmental functions were consistent with the top significant transcription factor motifs (Data S2). The most significantly (q < 0.01) enriched HOMER motifs were HEB (TCF12) for female placenta (Figure 2E), SMAD4 for male placenta (Figure 2F), SCL (TAL1) for female brain (Figure 2G), and PTF1A for male brain (Figure 2H). Among the top 10 motifs for the different pairwise comparisons, HIF1B (ARNT) and PTF1A were present in three, while AMYB, HEB (TCF12), HIC1, MYB, NANOG, SCL (TAL1), TBX5, and THRB were present in two comparisons. In a separate analysis of human-methylation-sensitive transcription factor motif enrichments within the PCB DMRs (Yin et al., 2017), the top motifs were related to transcription factors involved in early development, and members of the hairy and enhancer of split (HES), HES-related with YRPW motif (HEY), and achaete-scute complex-like (ASCL) transcription factors families were shared between sexes and tissues (Figures S4E–S4H; Data S2). PCB DMRs from the placenta of both sexes and female brain were also enriched for multiple motifs from the cyclic AMP (cAMP) responsive element binding protein (CREB) family. Finally, PCB DMRs from the placenta of both sexes were also enriched for motifs involved in circadian rhythm (ARNTL and CLOCK).

### Prenatal PCB exposure DMRs are enriched for within CpG islands and bivalent chromatin

**Figure 3.**
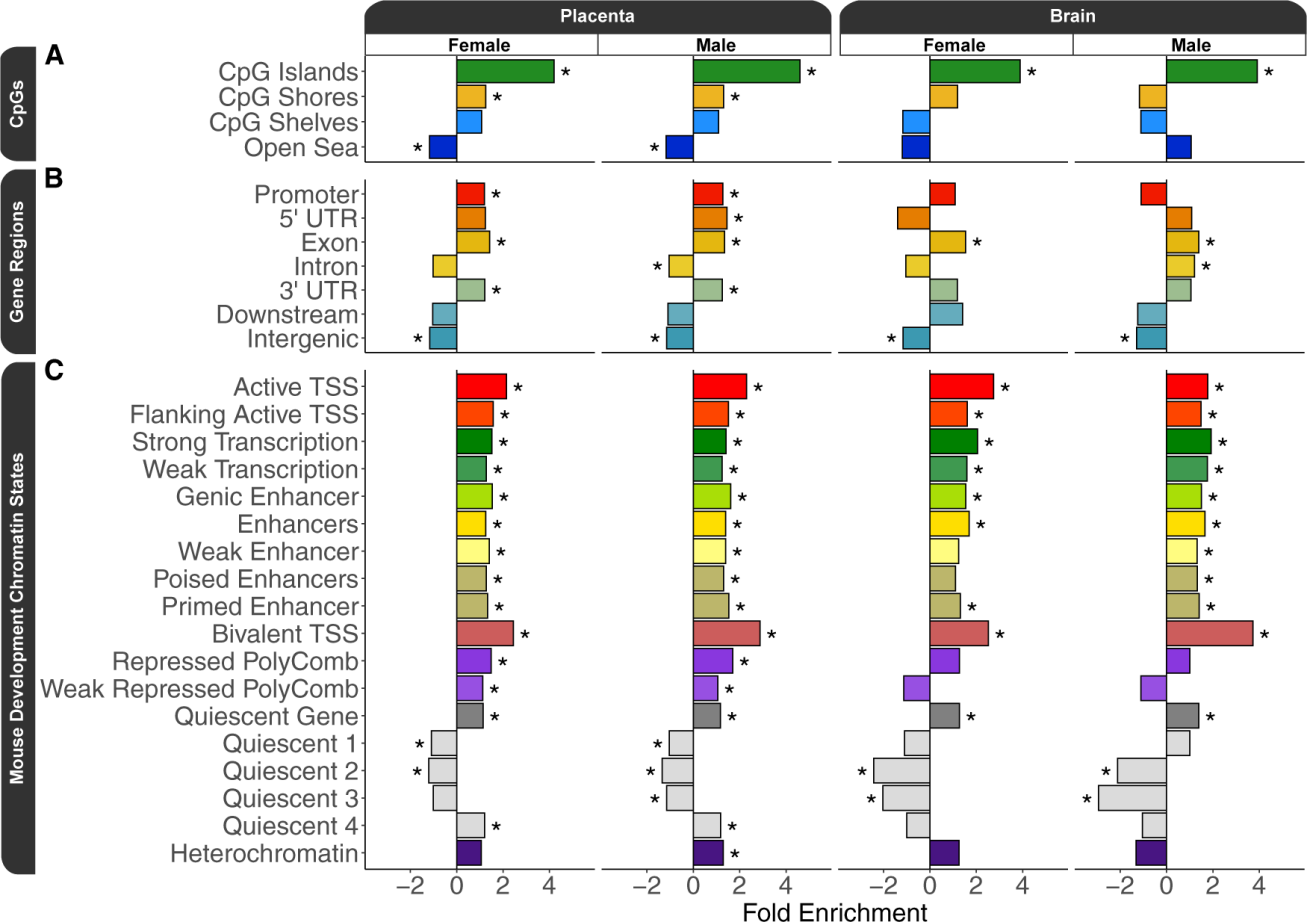
Annotation enrichment testing results for prenatal PCB exposure DMRs from sex-stratified pairwise comparisons of placenta and fetal brain (A) CpG annotation enrichments. (B) Gene region annotation enrichments. (C) Top developmental time point enrichments for mouse forebrain chromatin states. *q < 0.05.

To further test the hypothesis that PCB exposure resulted in methylation differences relevant to gene regulation, the PCB DMRs were tested for enrichment within annotated regions of the genome relative to their background regions. The first set of annotation enrichment testing was a tissue agnostic approach to examine CpG and gene region annotations (Data S2). Although only 1% (288 out of 28,628) of all PCB DMRs overlapped CpG islands (Data S2), PCB DMRs were significantly (q < 0.05) enriched within CpG islands for both sexes and both tissue sources (Figure 3A). However, only the placental PCB DMRs were significantly (q < 0.05) enriched within CpG shores but depleted within the open sea (Figure 3A). PCB DMRs were significantly (q < 0.05) enriched within gene bodies but depleted within intergenic regions (Figure 3B). Placental PCB DMRs differed from those in brain in that they were also enriched within promoters. Finally, PCB DMRs were tested for enrichment within an 18-chromatin-state model of mouse embryonic development, specifically forebrain tissue (Data S2; van der Velde et al., 2021). PCB DMRs from all pairwise comparisons were significantly (q < 0.05) enriched within transcription start site (TSS) regions marked by active and bivalent chromatin during at least one developmental time point, and bivalent TSS was the top enrichment (odds ratio >2.4; q < 0.003) overall (Figure 3C).

### The placenta and brain PCB exposure DMRs intersect at NDD genes and loci

In order to directly test the significance of overlap between placenta and fetal brain DMRs resulting from prenatal PCB exposure, the placenta and brain PCB DMRs were examined from both the genomic coordinate and gene-mapping perspectives. When overlapped by genomic coordinate, the placenta-brain overlapping PCB DMRs mapped to 20 genes in females and 23 in males (Table 2). Since a number of the placenta-brain DMRs correlated with each other, the overall correspondence between the placenta-brain PCB DMRs was summarized at the individual level by examining the correlation of their eigengenes (Figure S5). There was a trend for a positive correlation in females (R = 0.32; p = 0.14) and a significant correlation in males (R = 0.69; p = 0.0006), where this effect appeared to be primarily from the PCB-exposed mice from both sexes.

**Figure 4.**
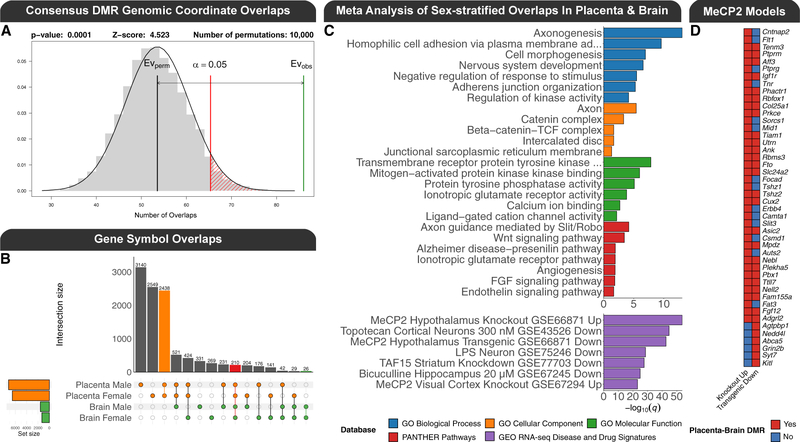
Overlaps between PCB exposure DMRs from placenta and fetal brain (A) Permutation analysis of the genomic coordinate enrichment of the sex-combined consensus fetal brain DMRs within the sex-combined consensus placenta DMRs. (B) UpSet plot of the gene symbol mapping overlaps for all pairwise DMR comparisons. (C) Top significant (q < 0.05) slimmed GO terms, PANTHER pathways, and GEO RNA-seq dataset enrichments from a meta p value analysis of gene symbol mappings from the sex-stratified overlaps of placenta and fetal brain. The GEO RNA-seq dataset enrichments are stratified by whether the genes were up- or down-regulated. (D) Heatmap of unique DMR gene symbol mappings that are shared between the female and male placenta-brain overlaps and repressed by MeCP2 in the hypothalamus of mouse models of Rett syndrome.

The impact of the placenta-brain overlapping PCB DMRs on gene expression differed by sex and tissue, and the gene regulatory profile was complex: some DMRs correlated with the expression of their gene mapping, many did not, and some correlated with the expression of many genes (Figure S6). To test for statistical significance of the overlaps, a permutation (n = 10,000) analysis of the genomic coordinate overlap based on region overlap, which randomly placed the DMRs across the entire genome while maintaining their size, uncovered a significant enrichment for the brain DMRs within the placenta DMRs for females (*Z* score = 2.9; empirical p = 0.006), males (*Z* score = 1.8; empirical p = 0.05), and the merging of regions by tissue to produce consensus DMRs (*Z* score = 4.5; empirical p = 0.0001; Figure 4A). A similar result (1.4-fold enrichment; empirical p = 0.0001) was observed in an independent approach that analyzed the nucleotide overlap of the consensus DMRs through random sampling (n = 10,000) of background regions, which had similar genomic context (CpG content and length), and this placenta-brain enrichment was also significant when the DMRs were stratified by hypermethylation (2.4-fold enrichment; empirical p = 0.004) and hypomethylation (1.5-fold enrichment; empirical p = 0.03). When the DMRs from all pairwise comparisons were mapped to their nearest gene, 210 overlapped by gene symbol (Figure 4B; Data S4). In contrast, this level of overlap was not observed for the DEGs (Figure S7A), but the overlap between DMRs and DEGs was more pronounced in the placenta (Figure S7B; Data S4).

In order to leverage the statistical power of the sex-stratified analyses, a meta p value analysis was performed on the sex-stratified functional enrichment testing results of the DMR gene symbol overlaps between placenta and brain (Data S4). The top significant (q < 0.05) slimmed GO enrichments were primarily related to cell adhesion, neurodevelopment, metabolism, and cellular signaling (Figure 4C). Among the top significant (q < 0.05) PANTHER pathways were axon guidance mediated by Slit/Robo, Wnt signaling, and the ionotropic glutamate receptor pathway. In addition to gene functions, this meta-analysis tested the DMR gene symbol overlaps for enrichments with 651 RNA-seq disease and drug signature datasets deposited in GEO, which were stratified by direction. The top significant (q < 0.05) enrichments were from studies of brain or neuronal responses to stimuli and primarily related to genes repressed by MeCP2 in mouse models of Rett syndrome. There were 86 unique genes shared between both the male and female placenta-brain overlaps and the top GEO datasets (Figure S8), and 46 of these are from a study of two mouse models of Rett syndrome, specifically the genes repressed by MeCP2 in the hypothalamus (Figure 4D).

Next, we examined the relevance of the findings to humans by testing the PCB DMRs for enrichment within regions identified by human epigenome-wide association studies (EWAS). First, we tested the hypothesis that PCB exposure DMRs are enriched within differentially methylated CpG sites identified by the Infinium Methylation EPIC BeadChip in humans with PCB exposure (Curtis et al., 2020; Pittman et al., 2020). Since the human studies analyzed both sexes together, the sex-combined tissue-specific consensus DMRs were tested after being lifted over to the human genome. Only the consensus brain DMRs were significantly (*Z* score = 6.7; q = 0.001) enriched within sites associated with PCB levels in human peripheral blood samples (Curtis et al., 2020). Second, we tested the hypothesis that the PCB exposure DMRs were enriched within DMRs associated with NDDs identified from brain. We utilized three of our previously published NDD DMR datasets after updating them to be processed similarly, including placenta from male patients with idiopathic ASD in the MARBLES cohort, brain from male patients with chromosome 15q11.2–13.3 duplication syndrome (Dup15q syndrome) and high PCB levels, and brain from female patients with Rett syndrome (Dunaway et al., 2016; Vogel Ciernia et al., 2020; Zhu et al., 2019). The consensus brain PCB-associated DMRs were significantly enriched within idiopathic ASD placenta (*Z* score = 2.1; q = 0.04), Dup15q syndrome brain (*Z* score = 5.2; q = 0.0006), and Rett syndrome brain DMRs (*Z* score = 2.4; q = 0.03). The consensus placenta PCB-associated DMRs were also significantly enriched within the idiopathic ASD placenta (*Z* score = 2.6; q = 0.02), Dup15q syndrome brain (*Z* score = 5.8; q = 0.0006), and Rett syndrome brain (*Z* score = 4.7; q = 0.0008) DMRs.

## DISCUSSION

This epigenomic study builds on previous foundational studies by providing novel insight into the role of DNA methylation in PCB-associated developmental neurotoxicity and NDDs. First, by characterizing the overlapping DNA methylation profiles in fetal brain and placenta from the same mice, we have uncovered shared DMRs in both tissues that are associated with a cellular signaling pathway that we have previously shown to result in developmental neurotoxicity from PCB exposure (Wayman et al., 2012a). Second, we also demonstrated that the PCB DMRs are associated with a genome-wide profile related to the NDD/ASD Rett syndrome, which is caused by mutations in the gene encoding DNA methylation binding protein 2 (*MECP2*). Notably, altered methylation of *MECP2* in the cord blood of human infants has been correlated with prenatal PCB exposure (Eguchi et al., 2019). We observed a significant overlap between the mouse placenta and brain consensus PCB DMRs with DMRs identified in human Rett syndrome brain. Furthermore, genes repressed by MeCP2 in mouse models were the top enrichments for the gene overlaps between placenta and brain (Chen et al., 2015). Together, these results demonstrate that placenta contains a subset of DMRs that overlap fetal brain DMRs associated with developmental neurotoxicity and NDDs.

To expand on the above summary, PCBs can be divided into two categories based on their mechanisms of toxicity: non-dioxin-like (NDL), which were represented by 11 of the 12 congeners in the MARBLES mixture, and dioxin like (DL), which were represented by the third most abundant congener in the mixture: PCB-118. While NDL and DL PCBs act through distinct mechanisms, there are similarities in that developmental exposure to NDL and DL PCBs has been shown to decrease levels of thyroid hormone in maternal serum (Bansal et al., 2005; Gauger et al., 2004; Giera et al., 2011; Zoeller, 2007). Thyroid hormone receptor beta (THRB) was among the top 10 transcription factor motifs enriched within placenta and brain PCB DMRs in females, with lower ranked enrichments in males. DL PCBs differ from NDL PCBs in that their primary mechanism involves binding to the aryl hydrocarbon receptor (AHR), which is then bound by the aryl hydrocarbon receptor nuclear translocator (ARNT), also known as hypoxia-inducible factor 1β (HIF1B), and translocated to the nucleus to activate genes involved in xenobiotic metabolism, such as cytochrome P450s (Calò et al., 2014; Kim et al., 2015; Klinefelter et al., 2018; Matsushita et al., 1993; Seok et al., 2017). HIF1B was one of the top 10 transcription factor motifs enriched in PCB DMRs from female placenta, male placenta, and female brain and had a lower ranked enrichment in male brain. Furthermore, a cytochrome P450 (*CYP2E1*) was one of the top ASD-associated DMRs in placental samples from the MARBLES study (Zhu et al., 2019), which was the reference for the PCB congener mixture used in this study (Sethi et al., 2019). Genes encoding multiple additional members of the cytochrome P450 family were also present in the placental PCB DMRs from our current study. Together, these results implicate known targets of DL PCBs and those shared with NDL PCBs; however, most of the DNA methylation differences observed with PCB exposure were related to the known mechanisms of the NDL PCBs.

Legacy and contemporary NDL PCBs predominately act through calcium-dependent mechanisms to alter synaptic connectivity (Klocke and Lein, 2020; Klocke et al., 2020). Neurodevelopmental terms related to synaptic connectivity and terms related to calcium ion binding were present in the top terms for the pairwise GO analyses as well as the GO terms for the gene overlaps between placenta and brain. The effects on calcium signaling are driven by legacy NDL PCBs activating signaling proteins on the cell membrane and endoplasmic reticulum, which were also enriched within both the pairwise and placenta-brain gene overlap GO analyses. At the cell membrane, PCBs activate NMDA receptors, a type of ionotropic glutamate receptor, and L-type voltage-gated calcium channels (Inglefield and Shafer, 2000; Llansola et al., 2009, 2010). Glutamate receptor functions were enriched within both GO molecular function and PANTHER pathway terms for the placenta-brain gene overlaps. PCBs also act on ryanodine and inositol 1,4,5-tris-phosphate receptors within the endoplasmic reticulum membrane (Inglefield et al., 2001; Pessah et al., 2010). Among all the membrane proteins targeted by legacy NDL PCBs, the most responsive are ryanodine receptors, which become sensitized (Klocke and Lein, 2020; Klocke et al., 2020; Panesar et al., 2020; Pessah et al., 2010). PCB 136 has been shown to sensitize ryanodine receptors and increase the frequency of spontaneous calcium oscillations in primary cultures of rat hippocampal neurons (Yang et al., 2014a). In our study, ryanodine receptor 1 (*Ryr1*) was the fifth-highest-ranked PCB DMR in female placenta and each pairwise PCB comparison contained more than one DMR mapping to a ryanodine receptor gene. The endoplasmic reticulum was also one of the top cellular component GO terms in male brain and was also consistent with the placenta-brain gene overlap cellular component GO terms.

The disruption to calcium signaling via the ryanodine receptor results in the deregulation of downstream developmental pathways. In primary rat hippocampal cultures, PCB 95 exposure has been shown to sensitize ryanodine receptor calcium channels, leading to increased calcium oscillations that activate the calcium/calmodulin-dependent protein kinase type 1 (CaMKI) and result in the CREB promoting transcription of *Wnt2* to ultimately stimulate dendritic growth and synaptogenesis (Lesiak et al., 2014; Wayman et al., 2012a, 2012b). The effects of the CaMKI signaling cascade on Wnt signaling appear to be the primary pathway for the genes that overlap between placenta and brain. The GO terms and pathways for the genes shared between placenta and brain primarily represent a cascade related to the cadherin pathway, which mediates calcium-ion-dependent cell adhesion. b-catenin is a subunit of the cadherin complex that functions as an intracellular signal transducer for the Wnt signaling pathway (Steinhart and Angers, 2018). The enrichment of the Wnt pathway is consistent with the placenta-brain genomic coordinate overlapped DMRs, specifically through *Daam2* in females, and through *Wnk2* in the top male brain DMRs (Lee and Deneen, 2012; Serysheva et al., 2013). Although some contemporary NDL PCBs are not as well studied, given their recent emergence, PCB 11 has been shown to effect dendritic arborization through a CREB-dependent mechanism in primary rat cortical neuron-glia co-cultures (Sethi et al., 2018). Therefore, while there are differences in the mechanisms of some legacy and contemporary NDL PCBs, they converge at CREB signaling. In our study, disruptions to CREB signaling are consistent with the mappings to *adenylate cyclase 1* (*Adcy1*) in the female placenta-brain genomic coordinate overlapped DMRs and *calcium/calmodulin-dependent protein kinase II, beta* (*Camk2b*) in the top male brain DMRs and the enrichment of CREB motifs within DMRs from female brain and placenta from both sexes. Overall, the results demonstrate that disruptions to known PCB-mediated signaling cascades are associated with differences in the brain and placental methylome.

The DNA methylation profile of prenatal PCB exposure also refines the effects on neurite outgrowth to the Slit/Robo signaling pathway. This pathway consists of the secreted Slit proteins and their receptors, the Roundabout (Robo) proteins. Although initially characterized for their role in axon guidance, members of Slit/Robo signaling are involved in dendritic growth and branching (Whitford et al., 2002). Slit/Robo signaling was the top ranked PANTHER pathway in the analysis of placenta and brain overlaps and among the top PANTHER pathways for all pairwise DMR comparisons, and roundabout binding was one of the top GO terms for female brain. Alterations to this signaling pathway are also consistent with the genomic coordinate overlaps between placenta and brain, specifically through *Slit1* in females and *Mid1* (*Midline 1*) in males (Liu et al., 2009; Whitford et al., 2002). Interestingly, many of the pathways that are downstream or cross-talk with Slit/Robo signaling in diverse cell types are associated with the prenatal PCB exposure DMRs, which include Wnt, phosphatidylinositol 3-kinase (PI3K)/AKT/mTOR, and transforming growth factor β (TGF-β) signaling (Blockus and Cheédotal, 2016). PCB 95 promotion of dendritic growth involves mTOR signaling in primary rat hippocampal neuron-glia co-cultures (Keil et al., 2018). The PI3K/AKT/mTOR pathway was associated with the female brain DMRs through the 1-phosphatidylinositol-3-kinase activity molecular function GO term and in male brain through the top DMR, which mapped to *cytosolic arginine sensor for mTORC1 subunit 2* (*Castor2*). Disruptions to TGF-β signaling are consistent with the results through its signal transducers: the Smad proteins. *Smad7* was one of the female placenta-brain genomic coordinate overlapping PCB DMRs, and Smad proteins were among the top transcription factor motif enrichments for male placenta and female brain. Thus, it appears that many intracellular signaling cascades that are downstream or cross-talk with Slit/Robo signaling are associated with the prenatal PCB exposure DMRs. In addition to a critical role in neurodevelopment, the above signaling cascade is also consistent with the anti-angiogenic effects of PCBs on placenta (Kalkunte et al., 2017). *Daam2*, a member of the Wnt signaling pathway, is involved in placental vascularization (Nakaya et al., 2020). The Slit/Robo pathway has also been implicated in placental angiogenesis (Bedell et al., 2005; Chen et al., 2016; Liao et al., 2012). The association with angiogenesis and vascularization is also apparent in the placenta-brain overlapping genes through the angiogenesis and endothelin signaling PANTHER pathway enrichments. Taken together, these findings show that the disrupted signaling pathways have distinct functions in both neurodevelopment and placental development.

Aside from being enriched within known signaling pathways, the genes mapping to PCB DMRs were strongly enriched for transcriptional dysregulation in neurodevelopmental disorders and neuronal drug responses from previously published datasets. The most prominent of the enrichments was for genes repressed by methyl-CpG binding protein 2 (MeCP2) in brain from mouse models of Rett syndrome (Chen et al., 2015; Gabel et al., 2015). One of these studies demonstrated that MeCP2 represses the expression of long genes enriched for ASD risk (Gabel et al., 2015). The NDD signature related to long genes and ASD risk is also apparent through an enrichment for genes down-regulated by topotecan, a topoisomerase inhibitor that reduces expression of many long genes associated with ASD in neurons (King et al., 2013). The disease and drug signature analysis also identified two other treatments that have been previously implicated in PCB exposure. Bicuculline is a GABA receptor agonist (Yu et al., 2015b), which has been shown to phenocopy the effects of PCB 95 on dendritic growth (Wayman et al., 2012a). There was also an immune signature associated with lipopolysaccharide (LPS) challenge in neurons (Srinivasan et al., 2016). This enrichment is consistent with the top female brain DMR mapping to *2900052N01Rik*, which shows high expression in B cells that have been stimulated by LPS (Wang et al., 2021), and through *LPS-responsive beige-like anchor* (*Lrba*), which was one of the top DMRs male brain. In addition, there were a number of immune genes in the top DMRs as well as the placenta-brain overlapped DMRs. Finally, there was a signature related to neurodegenerative diseases characterized by neuritic plaques and neurofibrillary tangles, specifically through a study of the role of *TAF15* in amyotrophic lateral sclerosis (ALS) and the Alzheimer disease-presenilin pathway from PANTHER (Kapeli et al., 2016). Ultimately, along with the genomic-coordinate-based enrichment of the consensus brain PCB-associated DMRs within differentially methylated sites from human lymphocytes with measured PCB exposures and the enrichment of the PCB-associated DMRs from both placenta and brain within DMRs identified from NDD placenta and brain, the prenatal PCB exposure DMRs identified in mouse showed a profile that is relevant to human NDDs.

The relevance of the PCB-associated DNA methylation profiles described in our study to human disorders appears consistent with the evolutionary conservation of developmental events. This is evidenced by the top chromatin state enrichment: bivalent TSS. Although all chromatin states are highly conserved between human and mouse, the bivalent TSS chromatin state, which represents 1.2% of the entire genome across all tissues and ~0.3% in a specific tissue, is substantially more evolutionarily conserved than the other 17 chromatin states (van der Velde et al., 2021). There are ~3,000 bivalent genes in each fetal tissue, which are poised for either activation and repression, and many of them are lineage-specific transcription factors that are repressed in the tissue assayed but expressed in others (Ngan et al., 2020; van der Velde et al., 2021). Given the tissue-specific nature of the identified bivalent chromatin state, future research into the effects of PCB exposure on DNA methylation profiles in placenta and brain would benefit from examining specific regions and cell populations through sorting or single-cell sequencing.

Finally, the overlaps between the DMRs and DEGs as well as the correlations between the placenta-brain overlapping PCB DMRs and gene expression highlight a complex gene-regulatory mechanism. DNA methylation not only has the potential to regulate transient gene expression profiles but also functions as a mark of past transcriptional alterations that can prime future responses (LaSalle et al., 2013; Treviño et al., 2020). In addition, DNA methylation is not always a repressive mark, as it is also associated with active transcription when in the gene body (Schroeder et al., 2015; Yang et al., 2014b). The larger number and size of the PCB DMRs detected in placenta, when compared with brain, may be due to the combination of its unique methylome that is characterized by global hypomethylation and PMDs as well as its role in functioning as the maternal-fetal interface, which is involved in detoxification. Ultimately, since placenta is a short-lived tissue that has characteristics of cancer, it may be more epigenetically responsive to environmental factors than other embryonic tissues.

Taken together, these findings demonstrate that a human-relevant PCB mixture results in placental DMRs that are also present in the developing brain, which is consistent with disruptions to cellular signaling pathways of relevance to both tissues. This suggests that the placenta, a typically discarded birth byproduct, contains a subset of PCB DMRs that overlap brain PCB DMRs and NDD DMRs. Although these PCB DMR profiles were obtained prior to birth (GD 18), future research would benefit from examining these regions at later time points, which include neonatal mouse brain and placenta, as well as later postnatal time points in brain and in response to later-life challenges. Finally, future research focused on DNA methylation profiling of human term placenta with measured PCB exposures, and maternal blood-derived, cell-free fetal DNA released from placental trophoblast (Alberry et al., 2007), could potentially lead to the development of informative biomarkers and enable early identification of prenatal exposures and early intervention of associated NDDs.

### Limitations of the study

A limitation of our study is that we did not examine the impact of genetic variation on the DMRs. It would be relevant for future studies to utilize a larger sample size of outbred mice. In addition, the impact of the DMRs on gene expression could be further investigated through a stimulus, such as an LPS challenge.

## STAR*METHODS

### RESOURCE AVAILABILITY

#### Lead contact

Further information and requests for resources and reagents should be directed to and will be fulfilled by the lead contact, Janine M. LaSalle (jmlasalle@ucdavis.edu).

#### Materials availability

This study did not generate new unique reagents.

#### Data and code availability

- Raw and processed sequencing data has been deposited at GEO and is publicly available as of the date of publication. The Accession number is listed in the key resources table.
- All original code has been deposited at GitHub and Zenodo and is publicly available as of the date of publication. The URL and DOI are listed in the key resources table.
- Any additional information required to reanalyze the data reported in this paper is available from the lead contact upon request.

### EXPERIMENTAL MODEL AND SUBJECT DETAILS

The PCB mixture formulated to mimic the 12 most abundant congeners identified from the serum of pregnant women in the ASD-enriched MARBLES cohort was prepared as previously described (Sethi et al., 2019). The PCB mixture consisted of the following congeners in differing proportions: PCB 28 (48.2%), PCB 11 (24.3%), PCB 118 (4.9%), PCB 101 (4.5%), PCB 52 (4.5%), PCB 153 (3.1%), PCB 180 (2.8%), PCB 149 (2.1%), PCB 138 (1.7%), PCB 84 (1.5%), PCB 135 (1.3%) and PCB 95 (1.2%). C57BL/6J dams (The Jackson Laboratory) aged 6 to 8 weeks were orally exposed to 1.0 mg/kg/d of the PCB mixture through diet (peanut butter) or vehicle (peanut oil in peanut butter) for at least 2 weeks before conception and during pregnancy, as previously described (Keil Stietz et al., 2021). Pregnant dams (*n_exposed_* = 4, *n_control_* = 5) were euthanized on gestational day 18 and whole brain and placenta were dissected from 44 fetuses, cut in half, and flash frozen. All protocols were approved by the Institutional Animal Care and Use Committee (IACUC) of the University of California, Davis.

### METHODS DETAILS

#### Nucleic acid extraction and high-throughput sequencing library preparation

Nucleic acids were extracted by homogenizing the same half of placenta and brain tissue using a TissueLyser II (Qiagen) followed by the AllPrep DNA/RNA/miRNA Universal Kit (Qiagen) according to the manufacturer’s instructions. For the low-pass WGBS libraries, DNA was sonicated to ~350 bp using a E220 focused-ultrasonicator (Covaris) and bisulfite converted using the EZ DNA Methylation-Lightning Kit (Zymo Research) according to the manufacturer’s instructions. Libraries were prepared via the Accel-NGS Methyl-Seq DNA Library Kit (Swift Biosciences) with the Methyl-Seq Combinatorial Dual Indexing Kit (Swift Biosciences) according to the manufacturer’s instructions. The pool of 88 libraries was sequenced on all 4 lanes of an NovaSeq 6000 S4 flow cell (Illumina) for 150 bp paired end reads, which yielded ~65 million unique aligned reads (~6X genome cytosine coverage) for each sample. For the RNA-seq libraires, RNA integrity (RIN > 7) was confirmed using a Bioanalyzer Eukaryotic Total RNA Nano Assay (Agilent). Libraries were prepared with the KAPA mRNA HyperPrep kit (Roche) and NEXTFLEX Unique Dual Index Barcodes (PerkinElmer). The pool of 88 libraries was sequenced on 1 lane of a NovaSeq 6000 S4 flow cell (Illumina) for 150 bp paired end reads, which yielded approximately 25 million uniquely mapped reads for each sample.

### QUANTIFICATION AND STATISTICAL ANALYSES

#### Bioinformatic analyses

The CpG_Me alignment pipeline (v1.4), which is based on Trim Galore (v0.6.5), FastQ Screen (v0.14.0), Bismark (v0.22.3), Picard (v2.18.4), and MultiQC (v1.9), was used to trim adapters and methylation bias, screen for contaminating genomes, align to the reference genome (mm10), remove duplicates, calculate coverage and insert size metrics, extract CpG methylation values, generate genome-wide cytosine reports (CpG count matrices), and examine quality control metrics (Ewels et al., 2016; Krueger and Andrews, 2011; Langmead and Salzberg, 2012; Laufer et al., 2020; Li et al., 2009; Martin, 2011; Wingett and Andrews, 2018). CpH and mitochondrial methylation levels were utilized to examine bisulfite conversion efficiency.

Since PCB exposure and NDDs are known to have substantial sex-specific effects (Keil et al., 2019; Sethi et al., 2017b), the primary analyses were stratified by sex. DMR calling and most downstream analyses and visualizations were performed via DMRichR (v1.6.1), which utilizes the dmrseq (v1.6.0) and bsseq (v1.22.0) algorithms (Hansen et al., 2012; Korthauer et al., 2018; Laufer et al., 2020). Background regions with similar genomic context to the DMRs (gene length and CpG content) were obtained from the first step of dmrseq for each pairwise comparison and utilized in most downstream enrichment testing. While it was not possible to model litter as a fixed or random effect in the DMR analyses for this data, litter was included as a fixed effect in the global methylation analyses. ComplexHeatmap (v2.2.0) was used to create the heatmaps (Gu et al., 2016). GOfuncR (v1.6.1) was used for genomic coordinate based gene ontology (GO) analyses, where DMRs were mapped to genes if they were between 5 Kb upstream to 1 Kb downstream of the gene body, and 1,000 random sets from samplings of the background regions were utilized for the FWER calculation (Grote, 2020; Prufer et al., 2007). Redundant GO terms were then removed based on semantic similarity using rrvgo (Sayols, 2020). HOMER (v4.10) was used to test for the enrichment of transcription factor motifs within the DMRs relative to the background regions using CpG% normalization and the exact sizes of the regions (Heinz et al., 2010). Memes (v1.0.0) was utilized to perform an Analysis of Motif Enrichment (AME), relative to background regions, through the MEME Suite with the Human Methylcytosine database and mm10 sequences (Bailey et al., 2009; McLeay and Bailey, 2010; Nystrom, 2021; Yin et al., 2017). ChIPseeker (v1.22.1) was used to obtain gene region annotations and gene symbol mappings (Yu et al., 2015a), promoters were defined as regions within 3 kb of the TSS, and DMRs were annotated to genes using the following prioritization order: Promoter > 5’ UTR > 3’ UTR > Exon > Intron > Downstream > Intergenic. Annotation based enrichment was performed using two sided Fisher exact tests and background regions, which was done through LOLA (v1.20.0) for the chromatin state enrichments (Sheffield and Bock, 2015), and the odds ratios were converted to fold enrichments for data visualization. Eigengenes were calculated from the first principal component of the individual smoothed methylation values for the placenta-brain DMRs. regioneR (v1.22.0) was utilized to perform permutation based genomic coordinate enrichment testing through a randomized region strategy with 10,000 permutations (Gel et al., 2016). GAT (v1.3.4) was used to perform nucleotide overlap enrichment testing through 10,000 random samplings of background regions (Heger et al., 2013). The RNA-seq alignment pipeline involved trimming adapters using Trim Galore (v0.6.5) followed by alignment to mm10 and gene count extraction using STAR (v2.7.3a) (Dobin et al., 2013), and an examination of QC metrics with MultiQC (v1.9). The DGE analysis utilized edgeR (v3.34.0) to filter the counts and dream (variancePartition v1.22.0) and limma-voom (v.3.48.1) to normalize the counts and fit a linear mixed model that included litter as a random effect, since it was not possible to model litter as a fixed effect for this dataset due to limma warning that some of the coefficients were not estimable (Hoffman and Roussos, 2021; Law et al., 2014; Robinson et al., 2010). ComplexUpset (v.1.3.1) was used to create UpSet plots of gene overlaps (Conwayet al., 2017; Krassowski, 2020; Lex et al., 2014). enrichR (v3.0) was used for gene symbol based GO, PANTHER pathway, and GEO RNA-seq disease and drug signature enrichment testing (Chen et al., 2013; Jawaid, 2021; Kuleshov et al., 2016; Xie et al., 2021). The overlap testing between WGBS and RNA-seq was based on gene symbol annotations.

## Supplementary Material

1

2

3

4

5

## Figures and Tables

**Table 1.** Top 10 DMRs from sex-stratified pairwise comparisons of prenatal PCB-exposed placenta and fetal brain

Chr	Region	Symbol	Name	Change	p value

Placenta: female					

9	intron	*5830454E08Rik*	*RIKEN cDNA 5830454E08 gene*	13%	9.0e-06
12	intron	*Ifrd1*	*Interferon-related developmental regulator 1*	14%	9.0e–06
X	promoter	*Msl3*	*MSL complex subunit 3*	−10%	1.0e–05
11	promoter	*Cpsf4l*	*Cleavage and polyadenylation specific factor 4-like*	12%	1.2e–05
7	exon	*Ryr1*	*Ryanodine receptor 1, skeletal muscle*	−9%	1.3e–05
2	intergenic	*Kcnj3*	*Potassium inwardly rectifying channel, subfamily J, member 3*	16%	3.3e–05
2	exon	*Jph2*	*Junctophilin 2*	−9%	3.3e–05
6	exon	*Zfp282*	*Zinc finger protein 282*	−12%	3.8e–05
5	exon	*Ints1*	*Integrator complex subunit 1*	13%	5.0e–05
18	intergenic	*Rit2*	*Ras-like without CAAX 2*	−25%	6.2e–05

Placenta: male					

11	exon	*Shroom1*	*Shroom family member 1*	21%	4.0e–06
5	intron	*Wasf3*	*WAS protein family, member 3*	−18%	6.0e–06
15	3′ UTR	*Cdh18*	*Cadherin 18*	−16%	1.0e–05
6	promoter	*Clstn3*	*Calsyntenin 3*	−15%	1.1e–05
2	intergenic	*Fsip2*	*Fibrous sheath-interacting protein 2*	−17%	1.1e–05
11	intergenic	*1700092K14Rik*	*RIKEN cDNA 1700092K14 gene*	−11%	1.5e–05
16	intron	*Erg*	*ETS transcription factor*	−10%	1.6e–05
17	intergenic	*4930470H14Rik*	*RIKEN cDNA 4930470H14 gene*	−14%	1.6e–05
4	intergenic	*Elavl2*	*ELAVlike RNA binding protein 1*	−17%	1.9e–05
10	intron	*Lama2*	*Laminin, alpha 2*	−14%	1.9e–05

Brain: female					

9	intergenic	*2900052N01Rik*	*RIKEN cDNA 2900052N01 gene*	−13%	2.9e–05
16	intron	*Mir99ahg*	*Mir99a and Mirlet7c-1 host gene*	−12%	4.7e–05
12	intergenic	*Begain*	*Brain-enriched guanylate kinase-associated*	−12%	9.4e–05
5	intron	*Vps37b*	*Vacuolar protein sorting 37B*	11%	1.4e–04
X	intergenic	*Bcor*	*BCL6 interacting corepressor*	−8%	1.4e–04
14	exon	*Gm30214*	*Predicted gene, 30214*	38%	2.0e–04
12	exon	*Elmsan 1*	*ELM2 and Myb/SANT-like domain containing 1*	−20%	2.2e–04
14	exon	*2900040C04Rik*	*RIKEN cDNA 2900040C04 gene*	−15%	2.6e–04
14	intron	*Wdfy2*	*WD repeat and FYVE domain containing 2*	−11%	2.7e–04
2	intron	*Myo3b*	*Myosin IIIB*	−11%	2.9e–04

Brain: male					

5	intergenic	*Castor2*	*Cytosolic arginine sensor for mTORC1 subunit 2*	−29%	2.7e–06
8	intron	*Tenm3*	*Teneurin transmembrane protein 3*	15%	8.0e–06
11	3′ UTR	*Camk2b*	*Calcium/calmodulin-dependent protein kinase II, beta*	−9%	1.9e–05
13	intron	*Wnk2*	*WNK lysine deficient protein kinase 2*	13%	1.9e–05
3	promoter	*Dapp1*	*Dual adaptor for phosphotyrosine and 3-phosphoinositides 1*	9%	3.2e–05
3	intron	*Lrba*	*LPS-responsive beige-like anchor*	−10%	6.7e–05
X	promoter	*Mid1*	*Midline 1*	−13%	8.0e–05
8	intergenic	*Maf*	*Avian musculoaponeurotic fibrosarcoma oncogene homolog*	−13%	9.1e–05
14	intron	*Extl3*	*Exostosin-like glycosyltransferase 3*	12%	1.0e–04
2	intron	*Cd44*	*CD44 antigen*	−12%	1.3e–04

**Table 2.** Sex-stratified genomic coordinate overlaps for prenatal PCB exposure DMRs from placenta and fetal brain

Chr	Start	End	Annotation	Symbol	Name	Placenta		Brain	
						Change	p value	Change	p value

Female									

X	168673277	168674466	promoter	*Msl3*	*MSL complex subunit 3*	−10%	1.0e–05	−6%	4.5e–02
8	125816527	125817293	intron	*Pcnx2*	*Pecanex homolog 2*	−10%	7.5e–04	−12%	2.8e–02
14	43122119	43122592	exon	*Gm10377*	*Predicted gene 10377*	7%	1.9e–03	−10%	3.7e–02
18	75382187	75384288	intron	*Smad7*	*SMAD family member 7*	−7%	5.9e–03	10%	4.7e–02
17	49559529	49560155	intron	*Daam2*	*Dishevelled associated activator*…	−11%	6.3e–03	12%	2.9e–02
X	71214809	71215506	promoter	*Mtml*	*X-linked myotubular myopathy gene 1*	−11%	6.5e–03	−8%	4.8e–02
14	103757379	103759757	intergenic	*Slainlos*	*SLAIN motif family, member 1, op*…	−6%	7.7e–03	−9%	2.7e–02
15	70554368	70555562	intergenic	*Gm19782*	*Predicted gene, 19782*	−8%	8.5e–03	−11%	1.3e–02
11	25661036	25667876	intron	*5730522E02Rik*	*RIKEN cDNA 5730522E02 gene*	−6%	1.3e–02	−12%	2.5e–02
9	65118461	65119611	exon	*Igdcc4*	*Immunoglobulin superfamily, DCC*…	−11%	1.3e–02	−9%	7.1e–03
2	128189766	128190007	exon	*Morrbid*	*Myeloid RNA regulator of BCL2L11*…	12%	1.9e–02	−9%	3.2e–02
19	41707495	41708350	intron	*Slit1*	*Slit guidance ligand 1*	−8%	2.4e–02	10%	2.2e–02
3	69912913	69913542	intergenic	*Sptssb*	*Serine palmitoyltransferase, sma*…	−9%	2.9e–02	10%	3.3e–03
1	74362604	74363214	promoter	*Catip*	*Ciliogenesis associated TTC17 in*…	−10%	3.1e–02	12%	4.8e–02
X	135796064	135796604	5’ UTR	*Gprasp1*	*G protein-coupled receptor assoc*…	9%	3.3e–02	10%	1.1e–02
5	33540194	33540690	intergenic	*Fam53a*	*Family with sequence similarity*…	−8%	3.3e–02	−7%	3.8e–02
11	7114091	7114722	intron	*Adcy1*	*Adenylate cyclase 1*	10%	3.6e–02	9%	1.7e–02
1	84086330	84088944	intron	*Pid1*	*Phosphotyrosine interaction doma*…	−7%	4.1e–02	−10%	4.4e–02
18	34340201	34341054	intergenic	*Srp19*	*Signal recognition particle 19*	−8%	4.1e–02	8%	5.9e–03
5	119778907	119779729	intergenic	*Tbx5*	*T-box 5*	−9%	4.5e–02	−12%	2.0e–02

Male									

Y	90761169	90762275	exon	*G530011O06Rik*	*RIKEN cDNA G530011O06 gene*	−10%	8.4e–05	−22%	1.8e–03
Y	90798875	90801260	intron	*Erdr1*	*Erythroid differentiation regula*…	−6%	1.9e–03	−14%	9.1e–04
X	169978999	169994536	promoter	*Mid1*	*Midline 1*	−7%	4.0e–03	−12%	1.1e–03
5	61928749	61931586	intergenic	*G6pd2*	*Glucose-6-phosphate dehydrogenase 2*	−11%	3.1e–03	11%	1.4e–02
7	24432388	24433272	promoter	*Irgc1*	*Immunity-related GTPase family*, …	−7%	5.8e–03	7%	1.2e–02
13	107438482	107439211	intergenic	*AI197445*	*Expressed sequence AI197445*	−11%	7.9e–03	−12%	1.8e–02
18	19500032	19503076	intergenic	*Dsc3*	*Desmocollin 3*	−10%	1.2e–02	10%	4.4e–02
7	134207540	134208460	intron	*Adam12*	*A disintegrin and metallopeptida*…	−8%	1.4e–02	10%	3.8e–02
3	86114243	86115189	exon	*Sh3d19*	*SH3 domain protein D19*	10%	1.8e–02	8%	3.5e–02
6	16549871	16550650	exon	*Gm36669*	*Predicted gene, 36669*	−8%	1.8e–02	6%	5.0e–02
5	117181539	117182559	intron	*Taok3*	*TAO kinase 3*	−9%	1.9e–02	11%	2.7e–02
4	51822204	51824406	intergenic	*C630028M04Rik*	*RIKEN cDNA C630028M04 gene*	−12%	2.0e–02	12%	3.7e–02
5	12988019	12989477	intergenic	*Sema3a*	*Sema domain, immunoglobulin doma*…	−11%	2.1e–02	−9%	1.5e–03
6	87285332	87286247	intron	*Antxr1*	*Anthrax toxin receptor 1*	−8%	2.2e–02	−12%	1.2e–02
5	53947218	53947797	intergenic	*Stim2*	*Stromal interaction molecule 2*	12%	2.2e–02	−11%	4.4e–02
X	97013515	97014853	intergenic	*Pgr15l*	*G protein-coupled receptor 15-like*	−17%	2.5e–02	10%	4.4e–02
13	39950021	39950769	intergenic	*Ofcc1*	*Orofacial cleft 1 candidate 1*	−7%	2.6e–02	9%	7.9e–03
12	82999424	83000586	intron	*Rgs6*	*Regulator of G-protein signaling 6*	−11%	3.5e–02	−10%	3.6e–02
17	29211657	29211879	exon	*Cpne5*	*Copine V*	−11%	3.9e–02	−12%	3.2e–02
13	73097375	73097948	intergenic	*Irx4*	*Iroquois homeobox 4*	9%	4.1e–02	11%	1.2e–02
17	75646796	75647687	intergenic	*Fam98a*	*Family with sequence similarity*…	−7%	4.5e–02	8%	9.0e–03
1	101834728	101837355	intergenic	*Gm20268*	*Predicted gene, 20268*	−10%	4.8e–02	8%	4.0e–02
5	89110706	89111345	intron	*Slc4a4*	*Solute carrier family 4 (anion e*…	−10%	4.9e–02	8%	3.0e–02

*Mid1* has multiple overlaps in the promoter and an intron, which have been summarized as the range of coordinates and averages of the change and p value.

**KEY RESOURCES TABLE:** 

REAGENT or RESOURCE	SOURCE	IDENTIFIER

Chemicals, peptides, and recombinant proteins		

PEBBLES PCB Mixture	Superfund Research Center at The University of Iowa	P42 ES013661

Critical commercial assays		

AllPrep DNA/RNA/miRNA Universal Kit	Qiagen	80224
EZ DNA Methylation-Lightning Kit	Zymo Research	D5031
Accel-NGS Methyl-Seq DNA Library Kit	Swift Biosciences	30096
Methyl-Seq Combinatorial Dual Indexing Kit	Swift Biosciences	38096
Bioanalyzer Eukaryotic Total RNA Nano Assay	Agilent	5067–1511
KAPA mRNA HyperPrep Kit	Roche	08098123702
NEXTFLEX Unique Dual Index Barcodes	PerkinElmer	NOVA-514150 NOVA-514152

Deposited data		

Raw sequencing data and processed count matrices	This paper	GEO: GSE180979
Custom code	This paper	https://github.com/ben-laufer/PCB-Placenta-and-Brain http://10.5281/zenodo.5037818

Experimental models: Organisms/strains		

Mice: C57BL/6J	The Jackson Laboratory	000664

Software and algorithms		

CpG_Me	Laufer et al. (2020)	https://github.com/ben-laufer/CpG_Me10.5281/zenodo.5030083
Trim Galore	Babraham Bioinformatics	https://www.bioinformatics.babraham.ac.uk/projects/trim_galore/
Cutadapt	Martin (2011)	https://cutadapt.readthedocs.io/en/stable/
FastQ Screen	Wingett and Andrews (2018)	https://www.bioinformatics.babraham.ac.uk/projects/fastq_screen/
Bismark	Krueger and Andrews (2011)	https://www.bioinformatics.babraham.ac.uk/projects/fastq_screen/
Bowtie 2	Langmead and Salzberg (2012)	http://bowtie-bio.sourceforge.net/bowtie2/index.shtml
Samtools	Li et al. (2009)	http://www.htslib.org
Picard	Broad Institute	https://broadinstitute.github.io/picard/
MultiQC	Ewels et al. (2016)	https://multiqc.info
R	https://www.r-project.org/	
DMRichR	Laufer et al. (2020)	https://github.com/ben-laufer/DMRichR10.5281/zenodo.5030057
Dmrseq	Korthauer et al. (2018)	https://bioconductor.org/packages/release/bioc/html/dmrseq.html
Bsseq	Hansen et al. (2012)	https://bioconductor.org/packages/release/bioc/html/bsseq.html
ComplexHeatmap	Gu etal. (2016)	https://jokergoo.github.io/ComplexHeatmap-reference/book/
GOfuncR	Grote (2020); Prüfer et al. (2007)	https://www.bioconductor.org/packages/release/bioc/html/GOfuncR.html
Rrvgo	Sayols (2020)	http://bioconductor.org/packages/release/bioc/html/rrvgo.html
HOMER	Heinz et al. (2010)	http://homer.ucsd.edu/homer/
Memes	Bailey et al. (2009); McLeay and Bailey (2010); Nystrom (2021); Yin et al. (2017)	http://www.bioconductor.org/packages/release/bioc/html/memes.html
ChIPseeker	Yu et al. (2015a)	https://bioconductor.org/packages/release/bioc/html/ChIPseeker.html
LOLA	Sheffield and Bock (2015)	https://bioconductor.org/packages/release/bioc/html/LOLA.html
STAR	Dobin et al. (2013)	https://github.com/alexdobin/STAR
edgeR	Robinson et al. (2010)	https://bioconductor.org/packages/release/bioc/html/edgeR.html
Limma-voom	Law et al. (2014)	https://bioconductor.org/packages/release/bioc/html/limma.html
variancePartition	Hoffman and Roussos (2021)	https://bioconductor.org/packages/release/bioc/html/variancePartition.html
regioneR	Gel et al. (2016)	https://bioconductor.org/packages/release/bioc/html/regioneR.html
GAT	Heger et al. (2013)	https://github.com/AndreasHeger/gat
ComplexUpset	Conway et al. (2017); Krassowski (2020); Lex et al. (2014)	https://cran.r-project.org/web/packages/ComplexUpset/index.html
enrichR	Chen et al. (2013); Jawaid (2021); Kuleshov et al. (2016); Xie et al. (2021)	https://cran.r-project.org/web/packages/enrichR/index.html

Other		

TissueLyser II	Qiagen	85300
E220 Focused-ultrasonicator	Covaris	500239

## Highlights

- Prenatal PCB exposure alters the DNA methylome of placenta and fetal brain in mice
- PCB DMRs are enriched for neurodevelopmental functions and bivalent chromatin
- A subset of DMRs overlap and are involved in Wnt and Slit/Robo signaling
- PCB DMRs are enriched for DMRs from human neurodevelopmental disorders
