# Supplementary material for: Placenta and fetal brain share a neurodevelopmental disorder DNA methylation profile in a mouse model of prenatal PCB exposure
Source: Cell Rep. Author manuscript; Available in PMC 2022 Mar 23. (PMC8941983; doi:10.1016/j.celrep.2022.110442)
Supplement: 1 [file NIHMS1785218-supplement-1.pdf]

Cell Reports, Volume *38*

Supplemental information

Placenta and fetal brain share a neurodevelopmental disorder DNA methylation profile in a mouse model of prenatal PCB exposure

Benjamin I. Laufer, Kari Neier, Anthony E. Valenzuela, Dag H. Yasui, Rebecca J. Schmidt, Pamela J. Lein, and Janine M. LaSalle

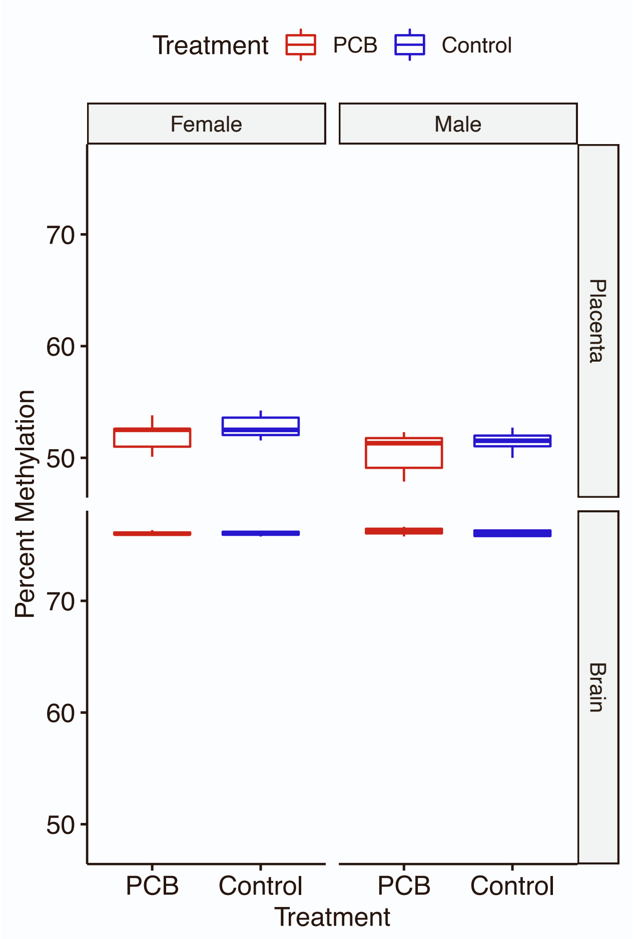

Figure S1: Box plots of smoothed individual global CpG methylation levels for placenta and brain from female and male PCB-exposed and matched control mice, Related to Figure 1.

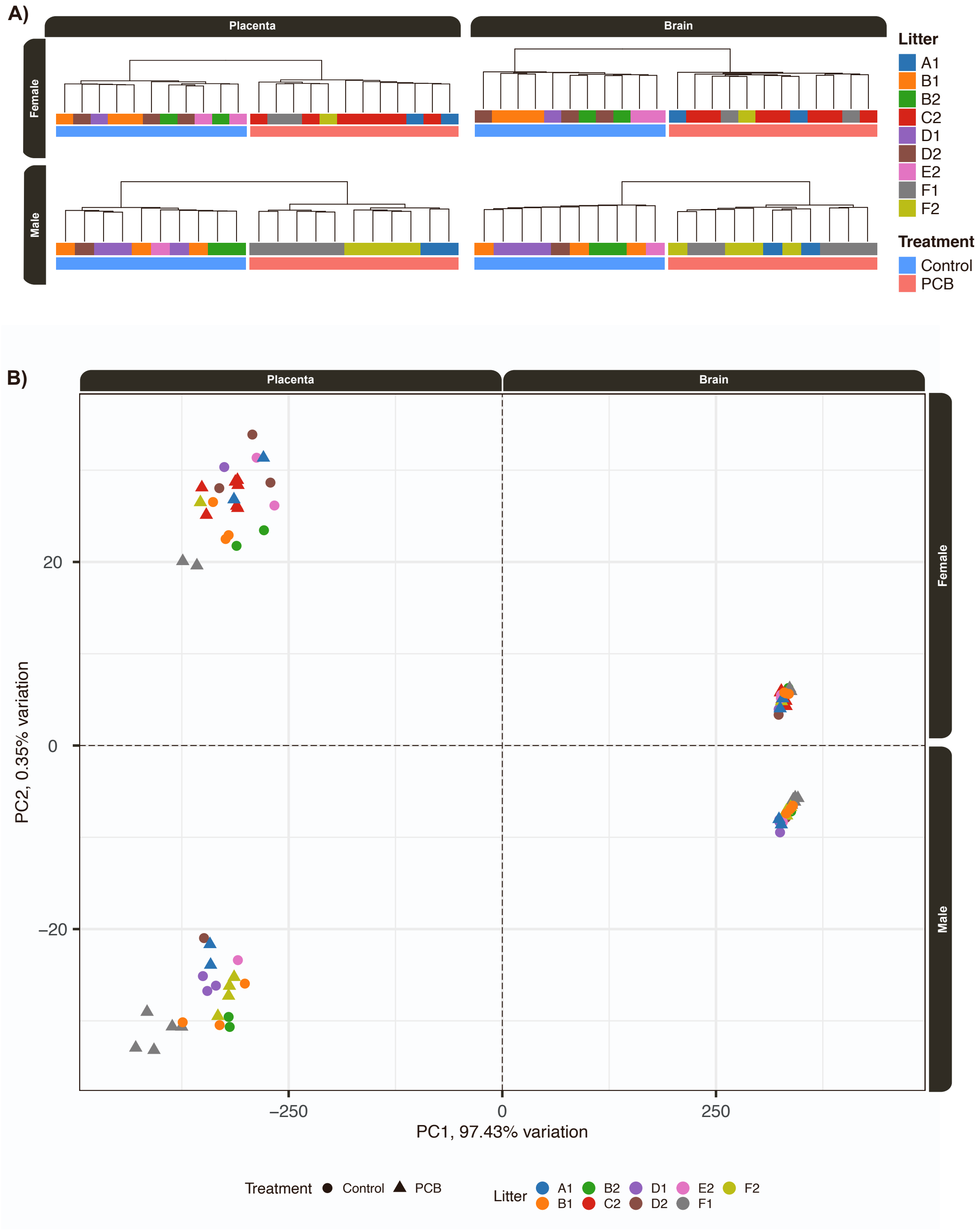

Figure S2: Litter effect analyses, Related to Figure 1. **A)** Expanded hierarchal clustering annotations for the Z-scores of regional smoothed methylation values for DMRs identified from sex-stratified pairwise analyses of placenta and brain. **B)** Principal component analysis (PCA) of smoothed individual methylation levels from 20 kb windows of the mouse genome. Regions with the lowest variance (bottom 10%) were removed from the analysis.

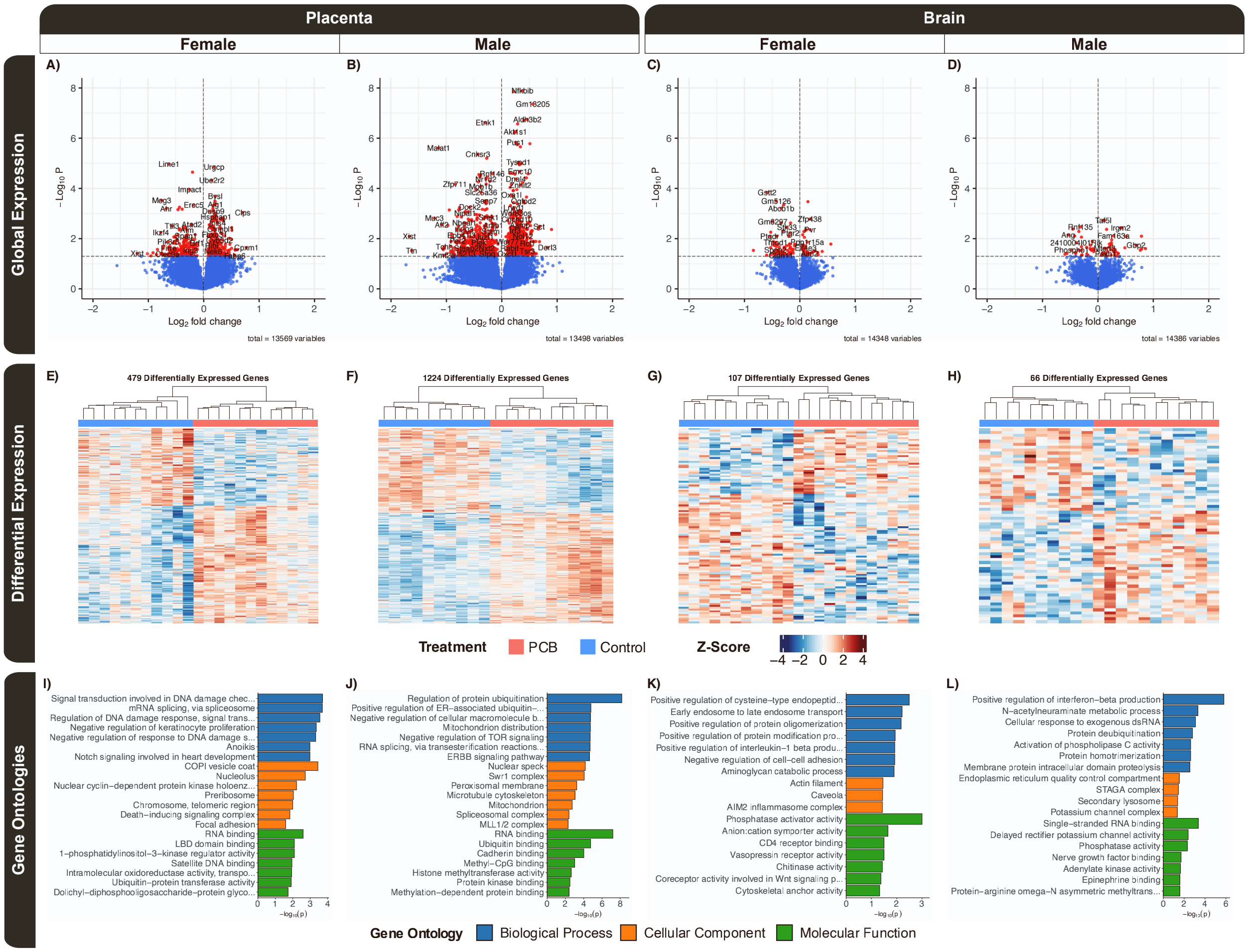

Figure S3: Sex-stratified RNA-seq gene expression profiles of placenta and fetal brain from mice with prenatal PCB exposure, Related to STAR Methods. Volcano plots of global and differential expression from **A)** female placenta, **B)** male placenta, **C)** female brain, and **D)** male brain. Blue dots indicate a non-significant gene and red dots indicate a DEG (nominal $p < 0.05$). Heatmaps of hierarchal clustering of the Z-scores of normalized expression values on the log2 scale for DEGs identified from pairwise comparisons of **E)** female placenta, **F)** male placenta, **G)** female brain, and **H)** male brain. Top slimmed significant ($p < 0.05$) GO enrichment results for DEGs from pairwise comparisons of **I)** female placenta, **J)** male placenta, **K)** female brain, and **L)** male brain. A total of 44 placenta and 44 fetal brain transcriptomes were generated from PCB-exposed GD18 males (n = 11) and females (n = 12) and matched vehicle control males (n = 10) and females (n = 11).

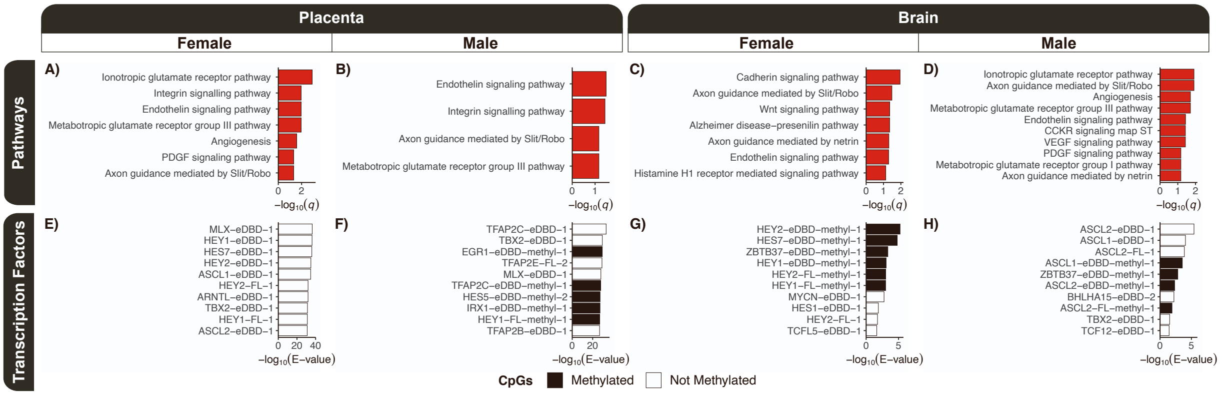

Figure S4: Expanded functional enrichment testing results for sex-stratified prenatal PCB exposure DMRs from placenta and fetal brain, Related to Figure 2. Top PANTHER pathway enrichment results for DMRs from pairwise comparisons of **A)** female placenta, **B)** male placenta, **C)** female brain, and **D)** male brain. Top human methylation sensitive transcription factor motif enrichment testing results for DMRs from pairwise comparisons of **E)** female placenta, **F)** male placenta, **G)** female brain, and **H)** male brain. The motif names indicate whether the transcription factor was full-length ("-FL") or an extended DNA-binding domain ("-eDBD"), if the CpGs were methylated ("-methyl"); and the number (starting with “-1”) distinguishes between multiple motifs.

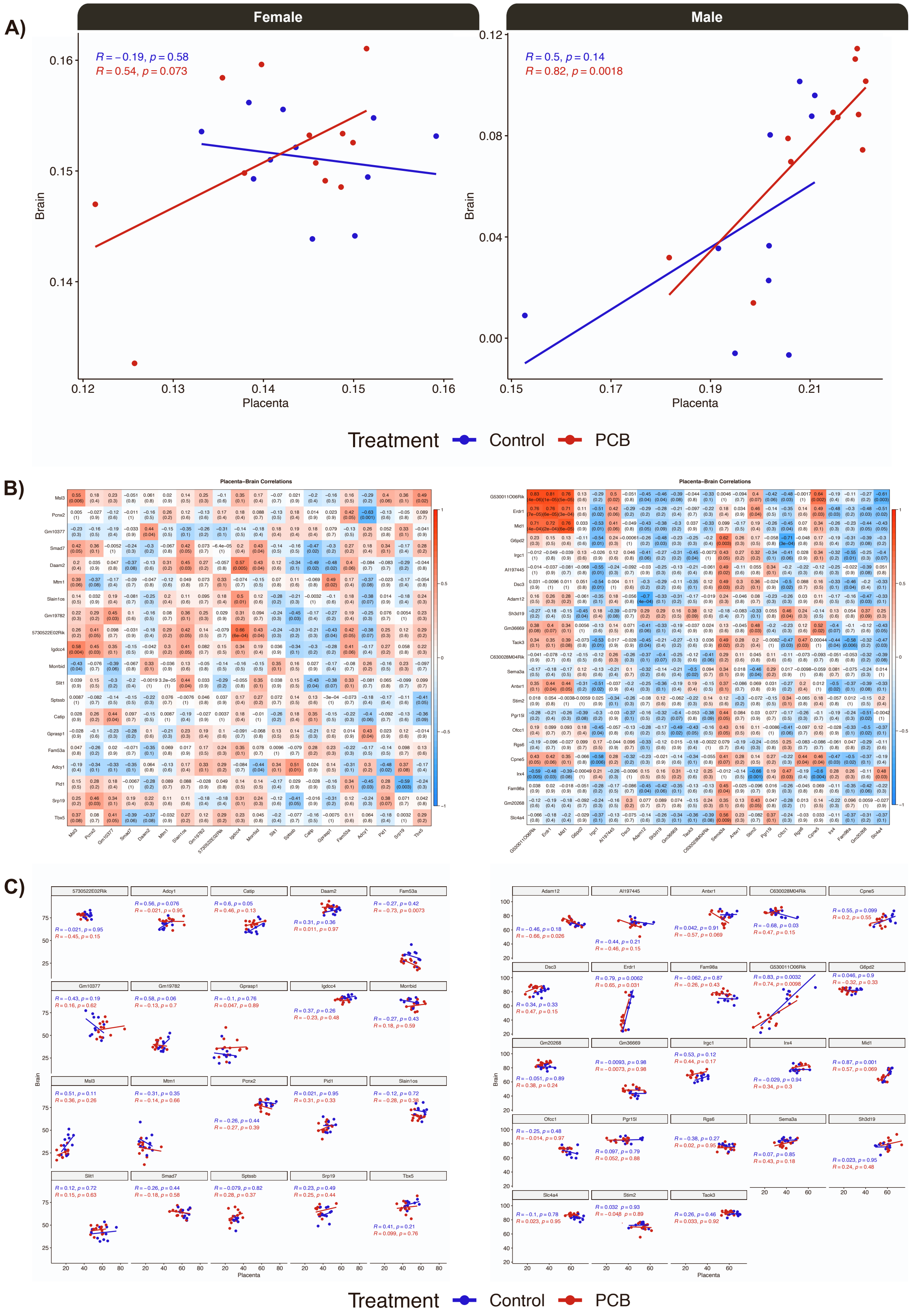
A)
Female
Male
R = −0.19, p = 0.58
R = 0.54, p = 0.073
R = 0.5, p = 0.14
R = 0.82, p = 0.0018
Brain
Placenta
B)
Placenta–Brain Correlations
Placenta–Brain Correlations
C)
Brain
Placenta
Treatment
Control
PCB

Figure S5: Correlation analyses of the sex-stratified placenta-brain overlapping PCB DMRs, Related to Table 2. **A)** Scatter plots of individual-level eigengene correlations stratified by treatment. **B)** Heatmaps of the correlations. **C)** Scatter plots of individual-level smoothed methylation value correlations stratified by treatment.

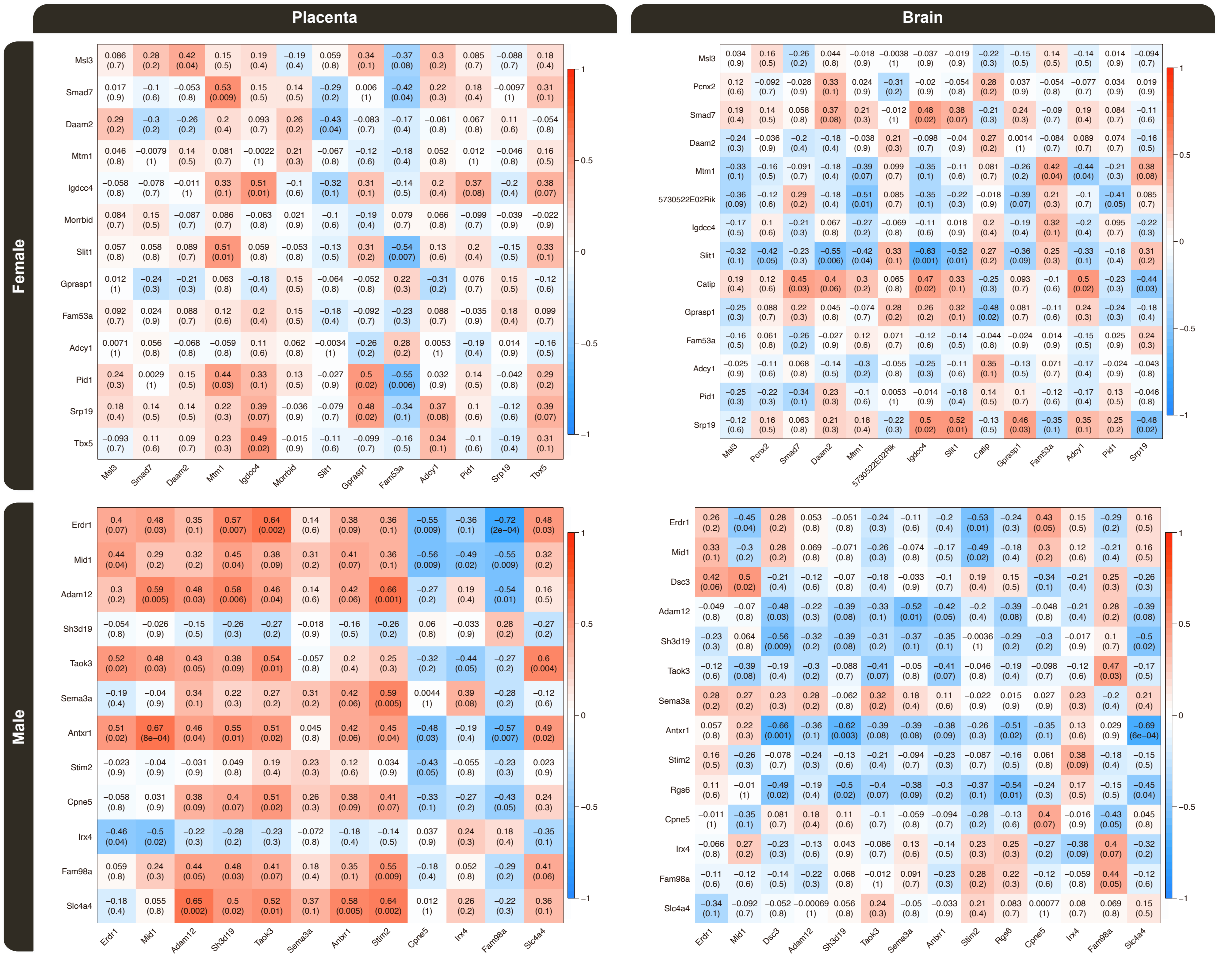

Figure S6: Correlation heatmaps of smoothed individual methylation values (y-axis) and normalized gene expression values on the log2 scale (x-axis) for the sex-stratified placenta-brain overlapping DMRs, Related to Table 2. DMR-transcript pairs were only included in the respective analysis if the transcript passed a detection filtering threshold. The parentheses contain the p-value for the correlation coefficient.

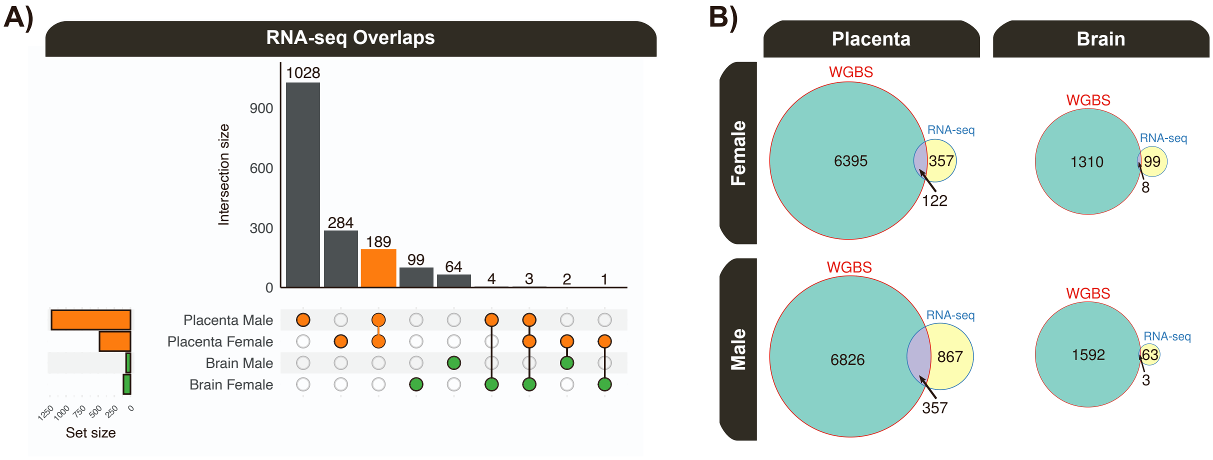

Figure S7: RNA-seq overlaps, Related to Figure 4 and STAR Methods. **A)** UpSet plot of sex-stratified DEG overlaps from pairwise comparisons of placenta and fetal brain. **B)** Venn diagrams of the overlap between DMRs from WGBS and DEGs from RNA-seq for sex-stratified pairwise comparisons of placenta and brain.

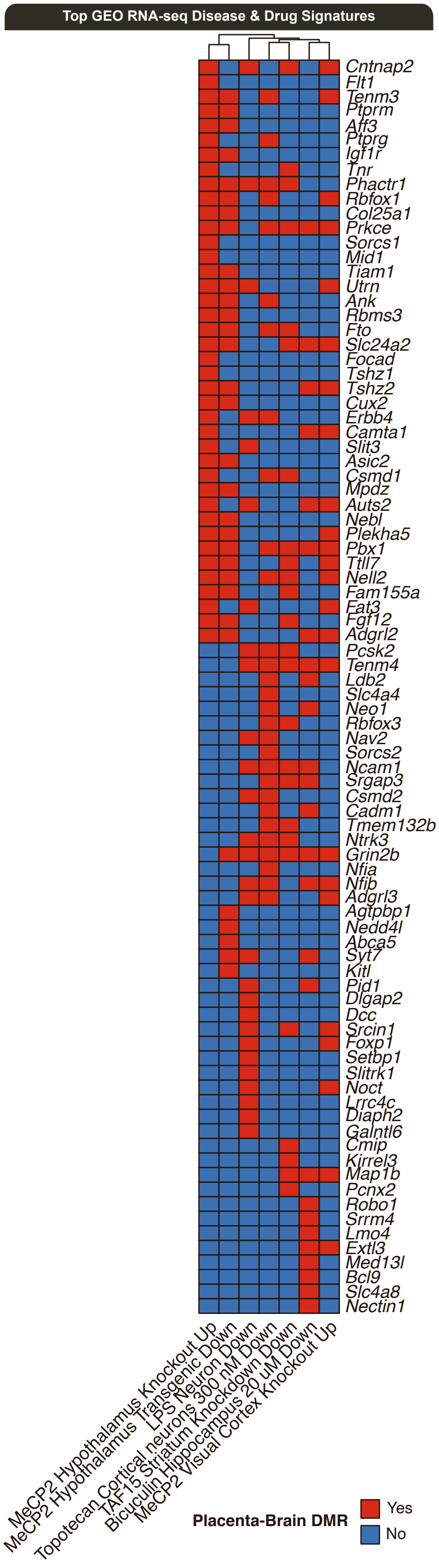

Figure S8: Heatmap of unique DMR gene symbol mappings that are shared between the female and male placenta-brain gene overlaps and the top enrichments from previously published RNA-seq datasets, Related to Figure 4.
